# Tris(2-methyl­piperidinium) tetra­chlorido­ferrate dichloride

**DOI:** 10.1107/S1600536812017151

**Published:** 2012-04-21

**Authors:** Qian Xu, Bao Cheng

**Affiliations:** a Ordered Matter Science Research Center, College of Chemistry and Chemical Engineering, Southeast University, Nanjing 211189, People’s Republic of China; b School of Chemical and Biomedical Engineering, YiChun University, YiChun 336000, People’s Republic of China

## Abstract

The asymmetric unit of the title salt, (C_6_H_14_N)_3_[FeCl_4_]Cl_2_, consists of a tetra­hedral tetra­chloro­ferrate anion, three independent 2-methyl­piperidinium cations and two chloride ions. All the piperidine rings adopt chair conformations. In the crystal, the organic cations and the free chloride anions are linked into chains parallel to the *a* axis by N—H⋯Cl hydrogen bonds.

## Related literature

For general background to ferroelectric compounds with metal-organic framework structures, see: Fu *et al.* (2009 ▶); Ye *et al.* (2006 ▶); Zhang *et al.* (2008 ▶, 2010 ▶). For ring puckering parameters, see: Cremer & Pople (1975 ▶).
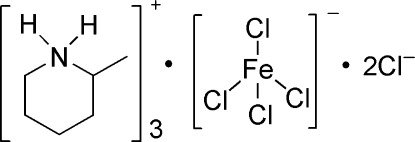


## Experimental

### 

#### Crystal data

(C_6_H_14_N)_3_[FeCl_4_]Cl_2_
*M* *_r_* = 569.10
Monoclinic, 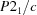

*a* = 10.443 (2) Å
*b* = 23.239 (5) Å
*c* = 14.494 (5) Å
β = 122.03 (2)°
*V* = 2982.0 (15) Å^3^
*Z* = 4
Mo *K*α radiation
μ = 1.05 mm^−1^
*T* = 293 K
0.28 × 0.26 × 0.21 mm

#### Data collection

Rigaku SCXmini diffractometer
Absorption correction: multi-scan (*CrystalClear*; Rigaku, 2005 ▶) *T*_min_ = 0.757, *T*_max_ = 0.809
30302 measured reflections
6848 independent reflections
2991 reflections with *I* > 2σ(*I*)
*R*_int_ = 0.116

#### Refinement

*R*[*F* ^2^ > 2σ(*F* ^2^)] = 0.077
*wR*(*F* ^2^) = 0.170
*S* = 1.03
6848 reflections
256 parameters
H-atom parameters constrained
Δρ_max_ = 0.49 e Å^−3^
Δρ_min_ = −0.34 e Å^−3^

### 

Data collection: *CrystalClear* (Rigaku, 2005 ▶); cell refinement: *CrystalClear*; data reduction: *CrystalClear*; program(s) used to solve structure: *SHELXS97* (Sheldrick, 2008 ▶); program(s) used to refine structure: *SHELXL97* (Sheldrick, 2008 ▶); molecular graphics: *DIAMOND* (Brandenburg & Putz, 2005 ▶); software used to prepare material for publication: *SHELXL97*.

## Supplementary Material

Crystal structure: contains datablock(s) I, global. DOI: 10.1107/S1600536812017151/rz2733sup1.cif

Structure factors: contains datablock(s) I. DOI: 10.1107/S1600536812017151/rz2733Isup2.hkl

Additional supplementary materials:  crystallographic information; 3D view; checkCIF report

## Figures and Tables

**Table 1:** Hydrogen-bond geometry (Å, °)

*D*—H⋯*A*	*D*—H	H⋯*A*	*D*⋯*A*	*D*—H⋯*A*
N3—H3*B*⋯Cl5	0.90	2.19	3.084 (4)	175
N3—H3*A*⋯Cl6	0.90	2.24	3.133 (4)	170
N2—H2*D*⋯Cl6^i^	0.90	2.26	3.118 (4)	160
N2—H2*C*⋯Cl5	0.90	2.26	3.156 (4)	171
N1—H1*B*⋯Cl6^ii^	0.90	2.28	3.183 (4)	178
N1—H1*A*⋯Cl5^iii^	0.90	2.21	3.105 (4)	174

Symmetry codes: (i) 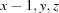; (ii) 
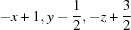
; (iii) 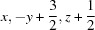.

**Fig. 1.**
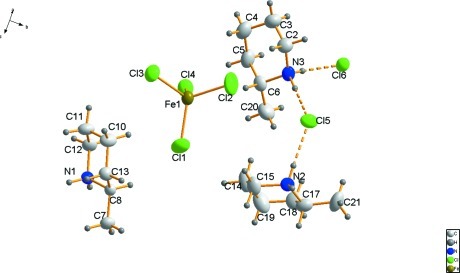
The molecular structure of the title compound with displacement ellipsoids drawn at the 30% probability level. Dashed lines indicate hydrogen bonds.

**Fig. 2.**
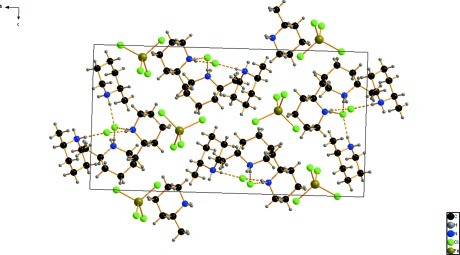
Crystal packing of the title compound viewed along the a axis. Dashed lines indicate hydrogen bonds.

## supplementary crystallographic information

### Comment

Dielectric constant measurements of compounds as a function of temperature is the basic method to find potential ferroelectric phase change materials (Fu *et al.*, 2009; Ye *et al.*, 2006; Zhang *et al.*, 2008; Zhang *et al.*, 2010). Unfortunately, the study carried out on the title compound indicated that the permittivity is temperature-independent, suggesting that there may be no dielectric disuniformity between 80 K to 350 K (m.p. 393–381 K). In this report the crystal structure of the title compound is reported.

An *ORTEP* diagram of the asymmetric unit of the title compound is shown in Fig. 1. In the tetrachloroferrate anion, the iron metal is coordinated in a tetrahedral geometry by four chloride anions with Fe—Cl distances ranging from 2.1698 (19) to 2.1909 (17) Å. In the three independent cations, the piperidine rings adopt a chair conformation with puckering parameters (Cremer & Pople, 1975) *Q* = 0.566 (5) Å, θ = 178.1 (6)° for ring N1/C8/C13/C19/C11/C12; *Q* = 0.551 (7) Å, θ = 1.7 (8)° for ring N2/C15/C14/C19/C18/C17; and *Q* = 0.561 (8) Å, θ = 1.7 (7)° for ring N3/C2–C6. The crystal structure is consolidated by an extensive network of intramolecular N—H···Cl hydrogen bonds (Table 1, Fig. 2) generating one-dimensional chains along the *a* axis.

### Experimental

An aqueous solution of 2-methylpiperidine (1.64 g, 20 mmol) and hydrochloric acid (10 mmol) was treated with FeCl_3_ (1.75 g, 10 mmol). After the mixture was churned for a few minutes, slow evaporation of the resulting solution yielded yellow crystals after a few days.

### Refinement

Hydrogen atom positions were placed at calculated positions and allowed to ride on their parent atoms, with C–H = 0.96–0.97 Å, N–H = 0.90 Å, and with *U*_iso_(H) = 1.2*U*_eq_(C, N) or or 1.5 *U*_eq_(C) for methyl H atoms.

### Crystal data

**Table:**

(C_6_H_14_N)_3_[FeCl_4_]Cl_2_	*F*(000) = 1196
*M**_r_* = 569.10	*D*_x_ = 1.268 Mg m^−^^3^
Monoclinic, *P*2_1_/*c*	Mo *K*α radiation, λ = 0.71073 Å
Hall symbol: -P 2ybc	Cell parameters from 6848 reflections
*a* = 10.443 (2) Å	θ = 2.7–27.5°
*b* = 23.239 (5) Å	µ = 1.05 mm^−^^1^
*c* = 14.494 (5) Å	*T* = 293 K
β = 122.03 (2)°	Block, yellow
*V* = 2982.0 (15) Å^3^	0.28 × 0.26 × 0.21 mm
*Z* = 4	

### Data collection

**Table:**

Rigaku SCXmini diffractometer	2991 reflections with *I* > 2σ(*I*)
Radiation source: fine-focus sealed tube	*R*_int_ = 0.116
Graphite monochromator	θ_max_ = 27.5°, θ_min_ = 3.0°
CCD_Profile_fitting scans	*h* = −13→13
Absorption correction: multi-scan (*CrystalClear*; Rigaku, 2005)	*k* = −30→30
*T*_min_ = 0.757, *T*_max_ = 0.809	*l* = −18→18
30302 measured reflections	2 standard reflections every 150 reflections
6848 independent reflections	intensity decay: none

### Refinement

**Table:**

Refinement on *F*^2^	Primary atom site location: structure-invariant direct methods
Least-squares matrix: full	Secondary atom site location: difference Fourier map
*R*[*F*^2^ > 2σ(*F*^2^)] = 0.077	Hydrogen site location: inferred from neighbouring sites
*wR*(*F*^2^) = 0.170	H-atom parameters constrained
*S* = 1.03	*w* = 1/[σ^2^(*F*_o_^2^) + (0.0479*P*)^2^ + 2.3248*P*] where *P* = (*F*_o_^2^ + 2*F*_c_^2^)/3
6848 reflections	(Δ/σ)_max_ = 0.001
256 parameters	Δρ_max_ = 0.49 e Å^−^^3^
0 restraints	Δρ_min_ = −0.34 e Å^−^^3^

### Special details

**Table:**

Geometry. All e.s.d.'s (except the e.s.d. in the dihedral angle between two l.s. planes) are estimated using the full covariance matrix. The cell e.s.d.'s are taken into account individually in the estimation of e.s.d.'s in distances, angles and torsion angles; correlations between e.s.d.'s in cell parameters are only used when they are defined by crystal symmetry. An approximate (isotropic) treatment of cell e.s.d.'s is used for estimating e.s.d.'s involving l.s. planes.
Refinement. Refinement of *F*^2^ against ALL reflections. The weighted *R*-factor *wR* and goodness of fit *S* are based on *F*^2^, conventional *R*-factors *R* are based on *F*, with *F* set to zero for negative *F*^2^. The threshold expression of *F*^2^ > σ(*F*^2^) is used only for calculating *R*-factors(gt) *etc*. and is not relevant to the choice of reflections for refinement. *R*-factors based on *F*^2^ are statistically about twice as large as those based on *F*, and *R*- factors based on ALL data will be even larger.

### Fractional atomic coordinates and isotropic or equivalent isotropic displacement parameters (Å^2^)

**Table:**

	*x*	*y*	*z*	*U*_iso_*/*U*_eq_	
C2	0.6651 (7)	0.8225 (3)	0.4704 (5)	0.0846 (18)	
H2A	0.5769	0.7996	0.4520	0.101*	
H2B	0.6341	0.8526	0.4162	0.101*	
C3	0.7818 (8)	0.7851 (3)	0.4700 (6)	0.104 (2)	
H3C	0.7380	0.7662	0.4000	0.125*	
H3D	0.8651	0.8088	0.4807	0.125*	
C4	0.8398 (8)	0.7407 (3)	0.5579 (6)	0.103 (2)	
H4A	0.9174	0.7180	0.5574	0.124*	
H4B	0.7581	0.7151	0.5439	0.124*	
C5	0.9046 (7)	0.7684 (3)	0.6683 (5)	0.092 (2)	
H5A	0.9372	0.7387	0.7234	0.110*	
H5B	0.9921	0.7913	0.6852	0.110*	
C6	0.7888 (6)	0.8063 (2)	0.6706 (5)	0.0697 (16)	
H6	0.7048	0.7820	0.6592	0.084*	
C7	0.0265 (6)	0.6181 (2)	0.8804 (4)	0.0723 (16)	
H7A	−0.0098	0.5852	0.9001	0.108*	
H7B	−0.0506	0.6470	0.8491	0.108*	
H7C	0.1146	0.6332	0.9441	0.108*	
C8	0.0663 (5)	0.6003 (2)	0.7987 (4)	0.0519 (12)	
H8	0.1115	0.6334	0.7843	0.062*	
C10	−0.0217 (6)	0.5604 (3)	0.6130 (4)	0.0774 (17)	
H10A	−0.1096	0.5455	0.5475	0.093*	
H10B	0.0161	0.5931	0.5928	0.093*	
C11	0.0982 (6)	0.5147 (3)	0.6639 (4)	0.0778 (17)	
H11A	0.0558	0.4801	0.6748	0.093*	
H11B	0.1320	0.5053	0.6149	0.093*	
C12	0.2305 (6)	0.5342 (2)	0.7709 (4)	0.0696 (16)	
H12A	0.3024	0.5029	0.8044	0.084*	
H12B	0.2807	0.5659	0.7592	0.084*	
N1	0.1796 (4)	0.55291 (16)	0.8445 (3)	0.0529 (10)	
H1A	0.2605	0.5647	0.9080	0.063*	
H1B	0.1392	0.5225	0.8587	0.063*	
C14	0.4725 (7)	0.8436 (3)	0.8182 (6)	0.111 (2)	
H14A	0.4796	0.8069	0.8526	0.134*	
H14B	0.5185	0.8393	0.7754	0.134*	
C15	0.3094 (6)	0.8596 (3)	0.7446 (5)	0.0861 (19)	
H15A	0.2600	0.8597	0.7854	0.103*	
H15B	0.2592	0.8315	0.6865	0.103*	
C17	0.3781 (6)	0.9647 (3)	0.7782 (5)	0.0782 (17)	
H17	0.3313	0.9685	0.8212	0.094*	
C18	0.5411 (6)	0.9466 (3)	0.8543 (5)	0.095 (2)	
H18A	0.5918	0.9463	0.8144	0.114*	
H18B	0.5913	0.9749	0.9121	0.114*	
C19	0.5566 (8)	0.8884 (4)	0.9041 (5)	0.115 (3)	
H19A	0.6626	0.8780	0.9477	0.138*	
H19B	0.5176	0.8898	0.9517	0.138*	
C20	0.8490 (8)	0.8379 (3)	0.7751 (5)	0.105 (2)	
H20A	0.9290	0.8631	0.7864	0.157*	
H20B	0.8872	0.8109	0.8340	0.157*	
H20C	0.7696	0.8602	0.7723	0.157*	
C21	0.3573 (7)	1.0197 (3)	0.7205 (6)	0.109 (2)	
H21A	0.4089	1.0181	0.6820	0.163*	
H21B	0.3980	1.0507	0.7723	0.163*	
H21C	0.2516	1.0262	0.6697	0.163*	
Cl1	0.2723 (2)	0.69890 (8)	0.69137 (18)	0.1180 (7)	
Cl2	0.2821 (3)	0.74617 (8)	0.45792 (17)	0.1363 (8)	
Cl3	0.24802 (18)	0.59654 (7)	0.49909 (14)	0.0918 (5)	
Cl4	0.59116 (16)	0.67217 (7)	0.67223 (14)	0.0900 (5)	
Cl5	0.46403 (15)	0.91536 (7)	0.56780 (12)	0.0779 (5)	
Cl6	0.95787 (14)	0.94390 (6)	0.60501 (12)	0.0673 (4)	
Fe1	0.34492 (8)	0.67911 (3)	0.57877 (6)	0.0621 (3)	
C13	−0.0665 (6)	0.5796 (2)	0.6919 (4)	0.0666 (15)	
H13A	−0.1400	0.6105	0.6589	0.080*	
H13B	−0.1143	0.5478	0.7055	0.080*	
N2	0.2980 (4)	0.91840 (17)	0.6970 (3)	0.0590 (11)	
H2C	0.3353	0.9165	0.6536	0.071*	
H2D	0.1998	0.9280	0.6551	0.071*	
N3	0.7290 (4)	0.84896 (17)	0.5811 (3)	0.0630 (12)	
H3A	0.8039	0.8732	0.5939	0.076*	
H3B	0.6564	0.8698	0.5811	0.076*	

### Atomic displacement parameters (Å^2^)

**Table:**

	*U*^11^	*U*^22^	*U*^33^	*U*^12^	*U*^13^	*U*^23^
C2	0.076 (4)	0.088 (5)	0.078 (4)	−0.004 (4)	0.033 (4)	−0.010 (4)
C3	0.126 (6)	0.095 (5)	0.113 (6)	0.004 (5)	0.078 (5)	−0.012 (5)
C4	0.100 (5)	0.079 (5)	0.130 (7)	0.013 (4)	0.060 (5)	−0.005 (5)
C5	0.077 (4)	0.068 (4)	0.114 (6)	0.011 (3)	0.040 (4)	0.013 (4)
C6	0.057 (3)	0.071 (4)	0.073 (4)	−0.013 (3)	0.029 (3)	0.004 (3)
C7	0.075 (4)	0.075 (4)	0.077 (4)	0.008 (3)	0.048 (3)	−0.006 (3)
C8	0.053 (3)	0.049 (3)	0.060 (3)	0.001 (2)	0.034 (3)	0.002 (2)
C10	0.067 (4)	0.099 (5)	0.053 (3)	0.013 (3)	0.023 (3)	−0.004 (3)
C11	0.081 (4)	0.093 (4)	0.064 (4)	0.010 (4)	0.042 (4)	−0.010 (3)
C12	0.063 (4)	0.080 (4)	0.078 (4)	0.023 (3)	0.046 (4)	0.009 (3)
N1	0.042 (2)	0.062 (3)	0.045 (2)	0.002 (2)	0.016 (2)	0.004 (2)
C14	0.075 (5)	0.094 (5)	0.133 (6)	0.002 (4)	0.034 (5)	0.043 (5)
C15	0.059 (4)	0.083 (4)	0.105 (5)	−0.011 (3)	0.036 (4)	0.014 (4)
C17	0.065 (4)	0.093 (5)	0.083 (4)	−0.004 (3)	0.043 (4)	−0.020 (4)
C18	0.061 (4)	0.110 (6)	0.084 (5)	−0.019 (4)	0.019 (4)	−0.013 (4)
C19	0.081 (5)	0.139 (7)	0.082 (5)	−0.021 (5)	0.014 (4)	0.031 (5)
C20	0.110 (5)	0.116 (6)	0.078 (5)	−0.002 (4)	0.042 (4)	0.013 (4)
C21	0.096 (5)	0.057 (4)	0.167 (7)	0.009 (4)	0.066 (5)	0.002 (4)
Cl1	0.1307 (16)	0.1068 (14)	0.169 (2)	−0.0277 (11)	0.1153 (16)	−0.0416 (13)
Cl2	0.1509 (19)	0.0924 (14)	0.1254 (16)	0.0199 (13)	0.0461 (15)	0.0492 (12)
Cl3	0.0892 (12)	0.0724 (10)	0.0997 (12)	−0.0215 (8)	0.0405 (10)	−0.0179 (9)
Cl4	0.0601 (9)	0.0925 (12)	0.1054 (13)	−0.0082 (8)	0.0358 (9)	0.0055 (9)
Cl5	0.0471 (8)	0.1127 (12)	0.0689 (9)	0.0165 (8)	0.0274 (7)	0.0055 (8)
Cl6	0.0538 (8)	0.0616 (8)	0.0963 (11)	−0.0050 (6)	0.0465 (8)	−0.0059 (7)
Fe1	0.0588 (5)	0.0532 (5)	0.0712 (5)	−0.0023 (4)	0.0324 (4)	0.0019 (4)
C13	0.055 (3)	0.079 (4)	0.059 (4)	0.010 (3)	0.026 (3)	0.005 (3)
N2	0.037 (2)	0.071 (3)	0.062 (3)	−0.002 (2)	0.021 (2)	−0.001 (2)
N3	0.039 (2)	0.064 (3)	0.086 (3)	0.003 (2)	0.033 (2)	0.007 (3)

### Geometric parameters (Å, º)

**Table:**

C2—C3	1.499 (8)	N1—H1B	0.9000
C2—N3	1.504 (6)	C14—C19	1.499 (9)
C2—H2A	0.9700	C14—C15	1.499 (8)
C2—H2B	0.9700	C14—H14A	0.9700
C3—C4	1.494 (8)	C14—H14B	0.9700
C3—H3C	0.9700	C15—N2	1.507 (6)
C3—H3D	0.9700	C15—H15A	0.9700
C4—C5	1.512 (8)	C15—H15B	0.9700
C4—H4A	0.9700	C17—C21	1.481 (8)
C4—H4B	0.9700	C17—N2	1.482 (6)
C5—C6	1.511 (7)	C17—C18	1.516 (8)
C5—H5A	0.9700	C17—H17	0.9800
C5—H5B	0.9700	C18—C19	1.501 (8)
C6—N3	1.482 (6)	C18—H18A	0.9700
C6—C20	1.489 (7)	C18—H18B	0.9700
C6—H6	0.9800	C19—H19A	0.9700
C7—C8	1.506 (6)	C19—H19B	0.9700
C7—H7A	0.9600	C20—H20A	0.9600
C7—H7B	0.9600	C20—H20B	0.9600
C7—H7C	0.9600	C20—H20C	0.9600
C8—N1	1.490 (5)	C21—H21A	0.9600
C8—C13	1.508 (6)	C21—H21B	0.9600
C8—H8	0.9800	C21—H21C	0.9600
C10—C11	1.505 (7)	Cl1—Fe1	2.1826 (19)
C10—C13	1.513 (7)	Cl2—Fe1	2.1698 (19)
C10—H10A	0.9700	Cl3—Fe1	2.1909 (17)
C10—H10B	0.9700	Cl4—Fe1	2.1860 (17)
C11—C12	1.499 (7)	C13—H13A	0.9700
C11—H11A	0.9700	C13—H13B	0.9700
C11—H11B	0.9700	N2—H2C	0.9000
C12—N1	1.487 (6)	N2—H2D	0.9000
C12—H12A	0.9700	N3—H3A	0.9000
C12—H12B	0.9700	N3—H3B	0.9000
N1—H1A	0.9000		
			
C3—C2—N3	109.8 (5)	C19—C14—C15	111.3 (6)
C3—C2—H2A	109.7	C19—C14—H14A	109.4
N3—C2—H2A	109.7	C15—C14—H14A	109.4
C3—C2—H2B	109.7	C19—C14—H14B	109.4
N3—C2—H2B	109.7	C15—C14—H14B	109.4
H2A—C2—H2B	108.2	H14A—C14—H14B	108.0
C4—C3—C2	111.1 (6)	C14—C15—N2	109.5 (4)
C4—C3—H3C	109.4	C14—C15—H15A	109.8
C2—C3—H3C	109.4	N2—C15—H15A	109.8
C4—C3—H3D	109.4	C14—C15—H15B	109.8
C2—C3—H3D	109.4	N2—C15—H15B	109.8
H3C—C3—H3D	108.0	H15A—C15—H15B	108.2
C3—C4—C5	111.1 (6)	C21—C17—N2	109.1 (5)
C3—C4—H4A	109.4	C21—C17—C18	115.0 (5)
C5—C4—H4A	109.4	N2—C17—C18	108.7 (5)
C3—C4—H4B	109.4	C21—C17—H17	108.0
C5—C4—H4B	109.4	N2—C17—H17	108.0
H4A—C4—H4B	108.0	C18—C17—H17	108.0
C6—C5—C4	111.0 (5)	C19—C18—C17	113.1 (5)
C6—C5—H5A	109.4	C19—C18—H18A	109.0
C4—C5—H5A	109.4	C17—C18—H18A	109.0
C6—C5—H5B	109.4	C19—C18—H18B	109.0
C4—C5—H5B	109.4	C17—C18—H18B	109.0
H5A—C5—H5B	108.0	H18A—C18—H18B	107.8
N3—C6—C20	108.4 (5)	C14—C19—C18	111.2 (5)
N3—C6—C5	109.9 (5)	C14—C19—H19A	109.4
C20—C6—C5	113.5 (5)	C18—C19—H19A	109.4
N3—C6—H6	108.3	C14—C19—H19B	109.4
C20—C6—H6	108.3	C18—C19—H19B	109.4
C5—C6—H6	108.3	H19A—C19—H19B	108.0
C8—C7—H7A	109.5	C6—C20—H20A	109.5
C8—C7—H7B	109.5	C6—C20—H20B	109.5
H7A—C7—H7B	109.5	H20A—C20—H20B	109.5
C8—C7—H7C	109.5	C6—C20—H20C	109.5
H7A—C7—H7C	109.5	H20A—C20—H20C	109.5
H7B—C7—H7C	109.5	H20B—C20—H20C	109.5
N1—C8—C7	109.7 (4)	C17—C21—H21A	109.5
N1—C8—C13	107.6 (4)	C17—C21—H21B	109.5
C7—C8—C13	114.0 (4)	H21A—C21—H21B	109.5
N1—C8—H8	108.4	C17—C21—H21C	109.5
C7—C8—H8	108.4	H21A—C21—H21C	109.5
C13—C8—H8	108.4	H21B—C21—H21C	109.5
C11—C10—C13	110.4 (5)	Cl2—Fe1—Cl1	112.09 (9)
C11—C10—H10A	109.6	Cl2—Fe1—Cl4	107.82 (8)
C13—C10—H10A	109.6	Cl1—Fe1—Cl4	108.26 (8)
C11—C10—H10B	109.6	Cl2—Fe1—Cl3	110.25 (8)
C13—C10—H10B	109.6	Cl1—Fe1—Cl3	109.40 (7)
H10A—C10—H10B	108.1	Cl4—Fe1—Cl3	108.95 (7)
C12—C11—C10	111.4 (5)	C8—C13—C10	112.6 (4)
C12—C11—H11A	109.4	C8—C13—H13A	109.1
C10—C11—H11A	109.4	C10—C13—H13A	109.1
C12—C11—H11B	109.4	C8—C13—H13B	109.1
C10—C11—H11B	109.4	C10—C13—H13B	109.1
H11A—C11—H11B	108.0	H13A—C13—H13B	107.8
N1—C12—C11	110.3 (4)	C17—N2—C15	114.9 (4)
N1—C12—H12A	109.6	C17—N2—H2C	108.6
C11—C12—H12A	109.6	C15—N2—H2C	108.6
N1—C12—H12B	109.6	C17—N2—H2D	108.6
C11—C12—H12B	109.6	C15—N2—H2D	108.6
H12A—C12—H12B	108.1	H2C—N2—H2D	107.5
C12—N1—C8	113.4 (4)	C6—N3—C2	113.8 (4)
C12—N1—H1A	108.9	C6—N3—H3A	108.8
C8—N1—H1A	108.9	C2—N3—H3A	108.8
C12—N1—H1B	108.9	C6—N3—H3B	108.8
C8—N1—H1B	108.9	C2—N3—H3B	108.8
H1A—N1—H1B	107.7	H3A—N3—H3B	107.7
			
N3—C2—C3—C4	55.4 (7)	N2—C17—C18—C19	−52.8 (7)
C2—C3—C4—C5	−57.2 (8)	C15—C14—C19—C18	−55.8 (8)
C3—C4—C5—C6	56.6 (7)	C17—C18—C19—C14	55.0 (8)
C4—C5—C6—N3	−54.6 (6)	N1—C8—C13—C10	−56.3 (5)
C4—C5—C6—C20	−176.1 (5)	C7—C8—C13—C10	−178.2 (4)
C13—C10—C11—C12	−54.0 (7)	C11—C10—C13—C8	55.8 (6)
C10—C11—C12—N1	54.8 (6)	C21—C17—N2—C15	−179.9 (5)
C11—C12—N1—C8	−58.2 (6)	C18—C17—N2—C15	54.0 (6)
C7—C8—N1—C12	−177.6 (4)	C14—C15—N2—C17	−56.2 (7)
C13—C8—N1—C12	57.8 (5)	C20—C6—N3—C2	179.7 (5)
C19—C14—C15—N2	55.1 (7)	C5—C6—N3—C2	55.2 (6)
C21—C17—C18—C19	−175.3 (6)	C3—C2—N3—C6	−55.6 (6)

### Hydrogen-bond geometry (Å, º)

**Table:**

*D*—H···*A*	*D*—H	H···*A*	*D*···*A*	*D*—H···*A*
N3—H3*B*···Cl5	0.90	2.19	3.084 (4)	175
N3—H3*A*···Cl6	0.90	2.24	3.133 (4)	170
N2—H2*D*···Cl6^i^	0.90	2.26	3.118 (4)	160
N2—H2*C*···Cl5	0.90	2.26	3.156 (4)	171
N1—H1*B*···Cl6^ii^	0.90	2.28	3.183 (4)	178
N1—H1*A*···Cl5^iii^	0.90	2.21	3.105 (4)	174

Symmetry codes: (i) *x*−1, *y*, *z*; (ii) −*x*+1, *y*−1/2, −*z*+3/2; (iii) *x*, −*y*+3/2, *z*+1/2.
